# Differential rotation in cholesteric pillars under a temperature gradient

**DOI:** 10.1038/s41598-020-73024-0

**Published:** 2020-10-14

**Authors:** Jun Yoshioka, Fumito Araoka

**Affiliations:** 1 RIKEN Center for Emergent Matter Science (CEMS), 2-1 Hirosawa, Wako, Saitama 351-0198 Japan; 2 Department of Physical Sciences, Ritsumeikan University, 1-1-1 Noji-Higashi, Kusatsu, Shiga 525-8577 Japan

**Keywords:** Soft materials, Fluid dynamics, Statistical physics, thermodynamics and nonlinear dynamics

## Abstract

Steady rotation is induced in cholesteric droplets dispersed in a specific liquid solvent under a temperature gradient. In this phenomenon, two rotational modes have been considered: (1) collective rotation of the local director field and (2) rigid-body rotation of the whole droplet structure. However, here we present another rotational mode induced in a pillar-shaped cholesteric droplet confined between substrates under a temperature gradient, that is, a differential rotation where the angular velocity varies as a function of the radial coordinate in the pillar. A detailed flow field analysis revealed that every pillar under a temperature gradient involves a double convection roll. These results suggested that the differential rotation in the cholesteric pillars was driven by the inhomogeneous material flow induced by a temperature gradient. The present experimental study indicates that the coupling between the flow and the director motion plays a key role in the rotation of the cholesteric droplets under the temperature gradient.

## Introduction

Typical rotating machines, such as windmills, water wheels and turbines, have a motion mode called the rigid-body rotation whose angular velocity is basically the same everywhere in the object around the centre of rotation. Unless this condition is satisfied, their essential structures are deformed and may not be maintained anymore. Thus, the rigid-body rotation is possible at least as long as the rotating object is undeformable, such as solids. However, rotations in many natural phenomena, such as whirlpools and tornados in fluid systems, show another rotation mode, that is, differential rotation, which has different angular velocities at different positional coordinates^1^. In contrast to the rigid-body rotation in solids, the differential rotation in fluids brings no problem of structural breakdown, because most natural fluids, such as water and air, have no physical structure. However, there are some exceptions, one of which can be found in astronomy. The galaxies, the well-known rotating systems, show differential rotations of stars, i.e. the angular velocity decreases as a function of the distance from the galactic centre^2–5^. Thus, a galaxy can be regarded macroscopically as a fluid. However, the galaxies are also known to possess a spiral structure^2,3^, which contradicts the basis of the above discussion. Intuitively, in such a case, the spiralling structure will increasingly wind or unwind as time passes. However, no such destructive change has been observed, and the spiral structure is always preserved. This paradox is called the winding dilemma and is yet unsolved even in the present astronomical physics. The key point is that galaxies are not simple fluids but complex fluids possessing a spiral structure, which is the only known prerequisite for the emergence of the winding dilemma. In other words, any complex fluid systems are interesting physical objects to observe and discuss such long-argued paradoxical phenomena.

In this study, we present the differential rotation induced in the liquid crystal (LC) system, one of the well-known complex fluid states in which physical structures can retain macroscopically^6^. Among the LCs, cholesteric (Ch) LCs with a helical structure are of extensive interest because there have been many reported dynamic behaviours, e.g. photovariable helical winding^7^, dynamic topological solitons^8–10^ or steady rotational motions in droplets^11–25^, upon various external stimuli, such as temperature gradient, electric field, material flow and light irradiation. Recently, highly stable and efficient rotation of Ch LC droplets under a temperature gradient have been reported in dispersion systems^12–19^. Although the detailed mechanism of the stable and efficient rotation in these systems is not yet clear, two main rotational modes have been suggested: one is the collective rotation of the local director and the other is the rigid-body rotation of the whole droplet^12–14,17–19^. However, in the present work, we report another rotational mode in Ch LC, that is, the differential rotation of flow induced in micron-sized cylindrical-pillar-shaped droplets (hereafter, LC pillars) of Ch LC.

## Results

### Rotational Flow in Ch LC Pillars

**Figure 1:**
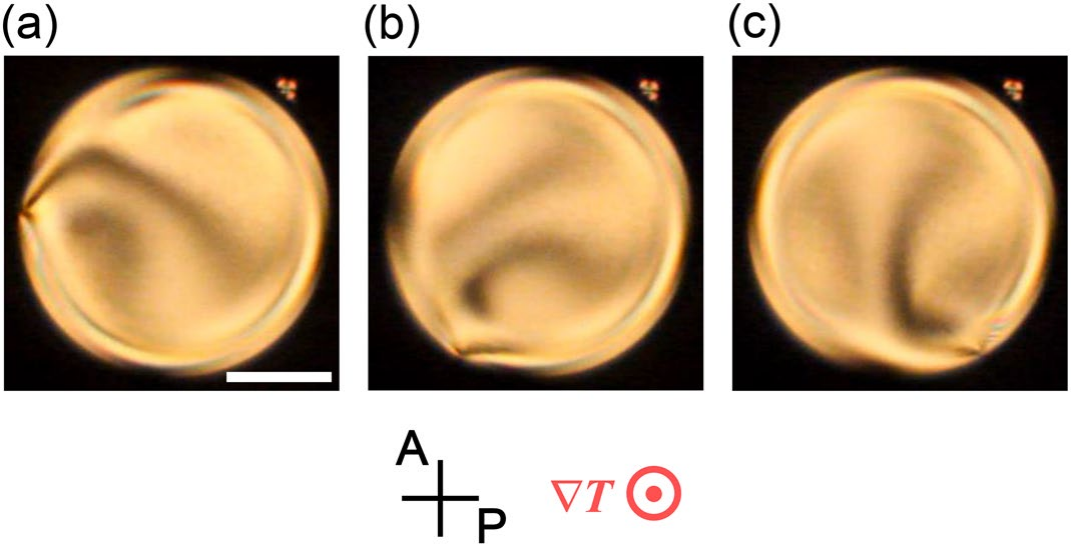
Rotation of the texture in the Ch LC pillar under a temperature gradient. The images were obtained by POM, and the polariser **P** and analyser **A** are shown in (**a**). The time interval between each image is 20 s. The white bar in (**a**) indicates 50 μm. The anchoring condition at the cell substrate was homeotropic. The concentration of the chiral dopant with respect to E8 was 0.15wt%, and the average temperature was 35 °C. The direction of the temperature gradient ∇*T* was perpendicular to the paper, and the applied heat flow was ~ 14.8 mW/mm^2^. A left-handed rotation was observed. The corresponding video is available in Supplementary Video 1 (SI1.mp4).

**Figure 2:**
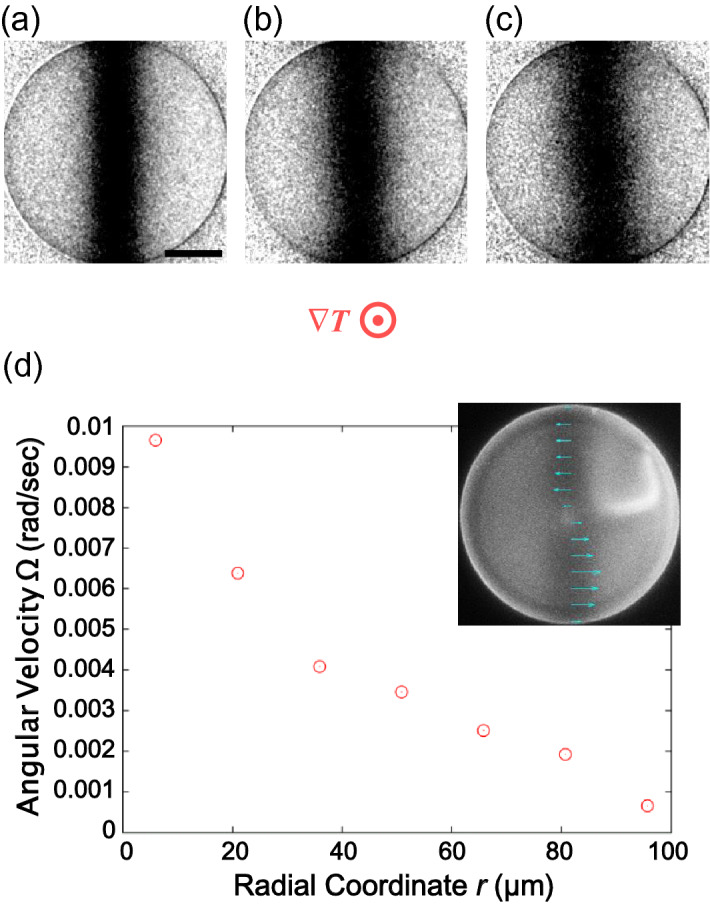
Rotational flow in the Ch LC pillar with a homeotropic anchoring condition. (**a**–**c**) are the fluorescence microscope images normalised by the ones before photobleaching. (**a**) Is the image just after the photobleaching, and the time interval between each image is 10 s. The black bar in (**a**) indicates 50 μm. The concentration of the chiral dopant with respect to E8 was 0.15wt%, and the average temperature was 35 °C. The direction of the temperature gradient ∇*T* is perpendicular to the paper, and the applied heat flow was ~ 14.8 mW/mm^2^. The inset of (**d**) is the distribution of the flow velocity component along the horizontal direction, shown together with the fluorescence microscope image without normalisation. The graph of (**d**) is the radial coordinate dependence of the angular velocity of the rotational flow Ω. The texture observed with the fluorescence microscopy (inset of **d**) showed the left-hand rotation, and its angular velocity ω was 0.040 rad/s.

LC pillars were prepared by confining the dispersion of a Ch LC in an isotropic fluorinated oligomer in LC cells with a homeotropic or homogeneous anchoring condition (see the method section). When the Ch LC pillar with a left-handed helix in a homeotropic cell (50 µm thick) was subjected to the temperature gradient parallel to the substrate normal, a stationary left-handed (counterclockwise) textural rotation, i.e. director rotation, was observed with polarizing microscopy (POM) as shown in Fig. 1 and Supplementary Video 1 (SI1.mp4). In such a situation, we found, by measuring the internal flow field in the Ch LC pillar, that the counterclockwise rotational flow exists around the central axis of the pillar, as shown in Fig. 2. Because its rotational speed was significantly slower than that of the director rotation measured by POM, we should consider that the director rotation is not driven by this rotational flow: the flow would be a result of the director rotation or just an independent phenomenon occurring in parallel with the director rotation. In particular, the angular velocity of the rotational flow depends on the radial coordinate $r$ on the Ch LC pillar, and thus the flow undergoes the differential rotation but not the rigid-body rotation. However, as the rotating pattern observed on POM (Fig. 1 and Supplementary Video 1) shows no remarkable change, the macroscopic director structure would be unchanged in spite of the differential rotation of the flow. This situation is just a resemblance of the aforementioned winding dilemma, the paradoxical phenomenon in the spiral galaxy.

**Figure 3:**
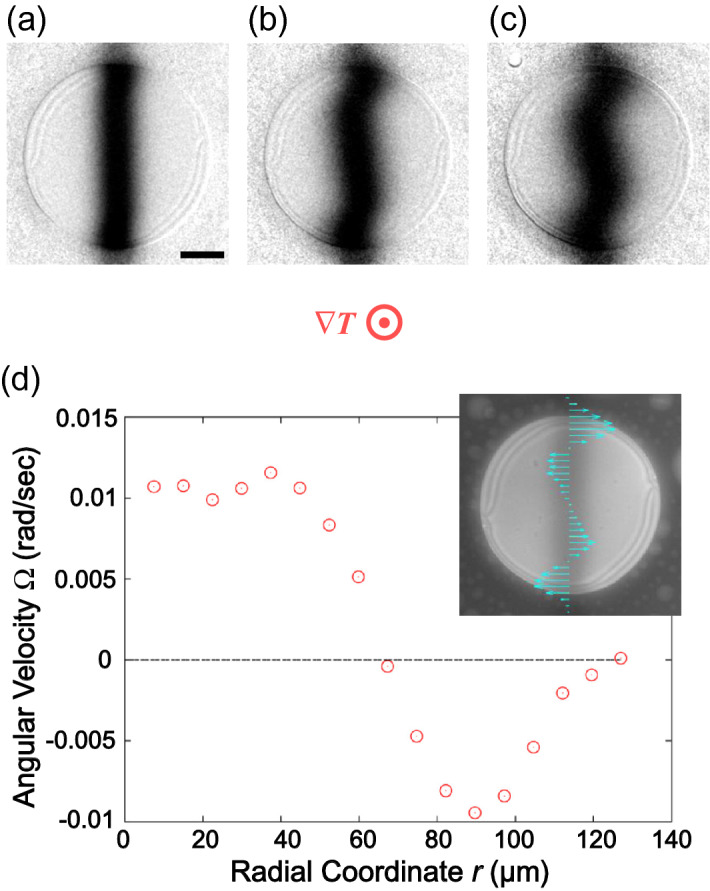
Rotational flow in the Ch LC pillar with a planar anchoring condition. (**a**–**c**) are the fluorescence microscope images normalised by the ones before photobleaching. (**a**) Is the image just after the photobleaching, and the time interval between each image is 10 s. The black bar in (**a**) indicates 50 μm. The concentration of the chiral dopant with respect to E8 was 0.5wt%, and the average temperature was 35 °C. The direction of the temperature gradient ∇*T* is perpendicular to the paper, and the applied heat flow was ~ 14.8 mW/mm^2^. The inset of (**d**) is the distribution of the flow velocity component along the horizontal direction, shown together with the fluorescence microscope image without normalisation. The graph of (**d**) is the radial coordinate dependence of the angular velocity of the rotational flow Ω.

The above observation of the director rotation was successful because the homeotropic anchoring does not fix the azimuthal angle of the surficial director. In contrast, in the cell with the homogeneous anchoring (20 µm thick), the director field was entirely fixed and indeed showed no rotation even under the temperature gradient (see Supplementary Video 2). However, interestingly, the rotational flow was still observed (Fig. 3). This fact suggests that the observed rotational flow occurs independently whether or not the director rotation exists, and it is not the consequential phenomenon resulting from the director rotation. Another remarkable feature of the rotational flow in the homogeneous cell is bidirectionality, i.e. the flow direction alters at a certain radial coordinate approximately $r = 65$ µm, as shown in Fig. 3d. Thus, the observed flow mode is still the differential rotation even in this case.

### Non-rotational Flow in N LC pillars

**Figure 4:**
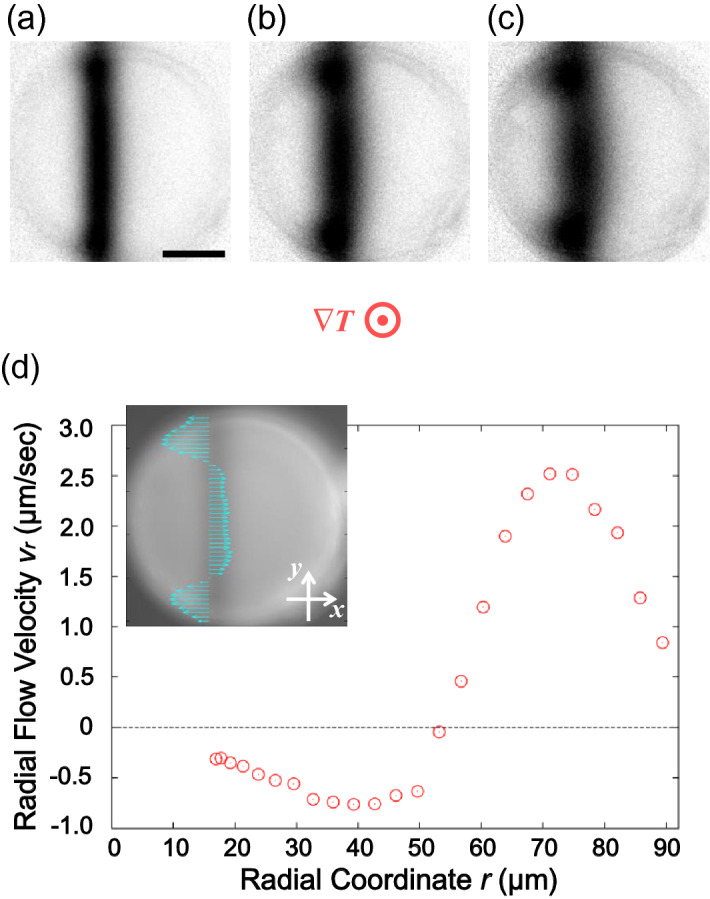
Measurement result of the radial flow in the N LC pillar near a high-temperature-side substrate. (**a**–**c**) are the fluorescence microscope images normalised by the ones before photo-bleaching. The black bar in (**a**) indicates 50 μm. No chiral dopant was added, and the average temperature was 35 °C. The direction of the temperature gradient ∇*T* is perpendicular to the paper, and the applied heat flow was ~ 14.8 mW/mm^2^. (**a**) is the image just after the photobleaching, and the time interval of each image is 5 s. The inset of (**d**) is the distribution of the flow velocity component along the horizontal direction $v_{x}$, shown together with the fluorescence microscope image without normalisation. The graph of (**d**) is the radial coordinate dependence of the radial flow velocity $v_{r}$, calculated by the assumption $v_{x} = v_{r} \cos \phi$. Here, a positive or negative value indicates the outward or inward flow, respectively.

To collect more useful information on the flow and its behaviour in the Ch LC pillars, we examined the flow field in the case of N LC. Because N LCs have no helical structure like Ch LCs, basically, the textural (director) rotation does not occur even under the temperature gradient. Thus, if the flow in the Ch LC pillar really occurs independently of the director rotation, then whether or not the flow still exists in N LC pillars should be examined. Accordingly, the same flow-field measurement was performed for an N LC pillar with a relatively thick LC cell (50 µm thick) to resolute the flows in the vicinity of the top and bottom cell surfaces. Figure 4 shows the results of the flow field analysis at the higher-temperature side in the cell with homeotropic anchoring. Because of absence of chirality, the rotational flow in this situation was not observed. Indeed, the observed bleaching pattern with a symmetric deformation (Fig. 4a–c) suggests no rotational flow but a radial flow. The radial flow velocity $v_{r}$ was determined with the relationship $v_{x} = v_{r} \cos \phi$, where $v_{x}$ is the *x*-component of the flow velocity and $\phi$ is the relative angle of the azimuthal coordinate (see Supplementary Method). The $v_{r}$ values are plotted in Fig. 4d, where the radial flow direction alters at approximately $r = 53$ µm. Different from the rotational flow in the Ch LC pillars, the alternation of the radial flow direction requires upward and downward flows or inward and outward flows.

**Figure 5:**
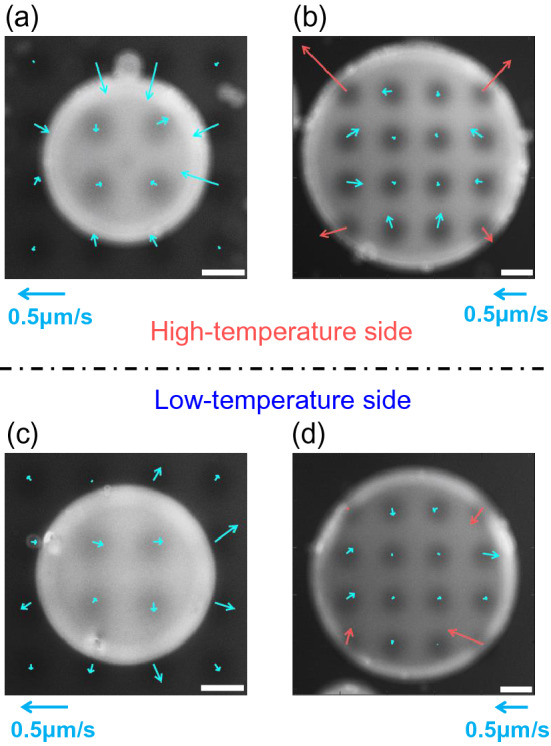
Flow fields in the N LC pillars near high- and low-temperature-side substrates. The white scale bars in (**a**–**d**) indicate 50 μm. The direction of the temperature gradient ∇*T* is perpendicular to the paper, and the applied heat flow was ~ 7.4 mW/mm^2^. The scale of the flow velocity is shown by the aqua arrow below each figure. The flow fields were obtained by the fluorescence photobleaching method. (**a**, **b**) show the field at the high-temperature side; (**c**, **d**), at the low-temperature side. In (**a**–**d**), the droplets with the sizes suitable for the respective measurements were chosen. In the solvent region of (**a**) or (**c**), the inward or outward flow was observed, respectively (see the flow in the eight lattice points nearest to the pillar). In the edge region of the pillar in (**b**) or (**d**), the outward or inward flow was observed respectively (see the flow in the four lattice points nearest to the pillar surface).

**Figure 6:**
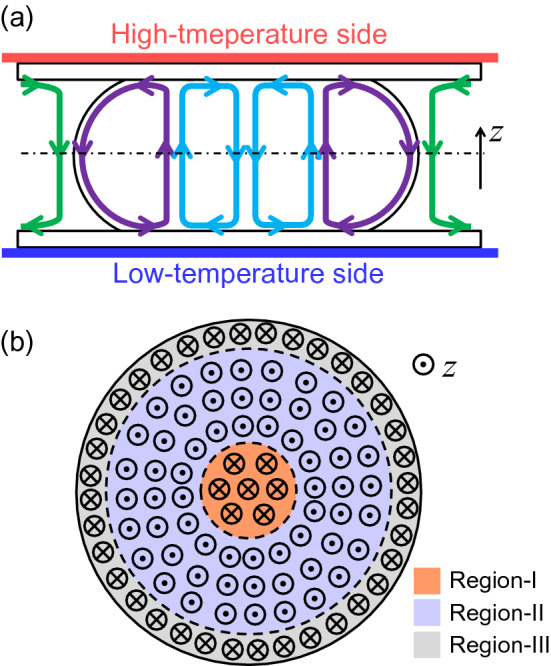
Schematic image of a double convective structure. (**a**) Is a side cross-sectional view, including the central axis of the pillar, and the flow is shown by the green, purple and aqua lines. (**b**) is the distribution of the z component of the flow velocity $v_{z}$ in the cross-sectional plane shown by the black chain line in (**a**). On the flow direction along the z direction (the sign of $v_{z}$), the pillar can be separated into three regions. In Regions-I and -III (orange and grey area), which are the central and edge regions, respectively, $v_{z}$ is negative, whereas $v_{z}$ is positive in Region-II (lavender area), which is the intermediate region between Regions-I and -III.

A flow field analysis using matrix spot illumination was performed for further mapping of the radial flow fields near the surfaces as shown in Fig. 5 where (a) and (b) are for higher temperature side, and 5(c) and (d) for lower temperature side. The flow directions at the corners (represented by the red arrows in Figs. 5(b) and (d)) show the outward and inward flows occurring at the edge region inside the N LC pillar on the higher- (Fig. 5b) and lower (Fig. 5d) temperature sides, respectively. By contrast, the solvent region just outside the N LC pillar shows the inward and outward flows in the higher (Fig. 5a)- and lower (Fig. 5c)-temperature sides, respectively, as shown by the aqua arrows. These results suggest that the upward/downward flows from the higher- to the lower-temperature sides exist around the bounding region between the N LC pillar and the surrounding solvent, as drawn by the green and purple lines in Fig. 6a. As already reported in refs. 18, 26 and 27, this flow is considered to be driven by a surface-tension gradient due to the temperature gradient. In this case, the Marangoni flow around the N LC pillar is the most likely candidate. In addition, the change in the radial flow direction dependent on the radial coordinate observed near the high-temperature side inside the pillar (Fig. 4d) suggests the existence of double convective flows in the N LC pillar, as described in Fig. 6a (see, purple and aqua lines). Consequently, the flow field in the cell depth direction (// *z*-axis) $v_{z}$ also alters inside the N LC pillar, as schematically drawn in Fig. 6b. We should consider that the double convective flow is also induced in the Ch LC pillars because the used N LC is actually the main component of the Ch LC and the addition of a small amount of a chiral dopant therein may hardly affect the generation of material flow. The flow fields induced in the N LC and Ch LC pillars were reported in refs. 26 and 27, and both looked similar when the temperature gradient was applied parallel to the cell substrate plane.

## Discussion

Considering all the above experimental results for the Ch and N LC pillars, the director rotation and flow rotations observed in the Ch LC pillars are driven by the vertical flows (// *z*-axis), i.e. these rotations are driven by the flow going through the helical structure along the helical axis, such as the mechanical turbine systems, similar to the discussions in refs. 18 and 22.

This section discusses the relationship between the vertical flow and the rotational phenomena in terms of fluid mechanics. Accordingly, we followed the method based on Onsager’s variational principle, recently introduced by M. Doi^28^. According to this theory, the time evolution of the system is determined by the minimised Rayleighian ${\Re }$, which is composed of the dissipation function *W* and the time differential of Frank’s free energy $\dot{F}$, that is, ${\Re } = W + \dot{F}$. However, $\dot{F}$ is actually negligibly small in the present case because the dynamic (time-dependent) director deformation is just scarcely observed under POM and hence not so significant. As for the dissipation function, the coupling terms between the flow and director fields can be described as^26–30^

1 $$W = \frac{1}{2}\beta_{1} \left( {e_{ij} n_{i} n_{j} } \right)^{2} + \frac{1}{2}\beta_{2} e_{ij}^{2} + \frac{1}{2}\beta_{3} \left( {e_{ij} n_{j} } \right)^{2} + \frac{1}{2}\gamma_{1} N_{i}^{2} + \gamma_{2} N_{i} e_{ij} n_{j} ,$$

2 $$e_{ij} = \frac{1}{2}\left( {\frac{{\partial v_{i} }}{{\partial x_{j} }} + \frac{{\partial v_{j} }}{{\partial x_{i} }}} \right),$$

3 $$N_{i} = \frac{{\partial n_{i} }}{\partial t} + v_{j} \frac{{\partial n_{i} }}{{\partial x_{j} }} - \frac{1}{2}\left( {\frac{{\partial v_{i} }}{{\partial x_{j} }} - \frac{{\partial v_{j} }}{{\partial x_{i} }}} \right)n_{j} ,$$

where *i* and *j* denote *x*, *y*, or *z*; $x_{i}$, $v_{i}$ and $n_{i}$ are the *i*-components of the positional vector, flow velocity vector and director field, respectively; $\beta_{1}$, $\beta_{2}$, $\beta_{3}$, $\gamma_{1}$ and $\gamma_{2}$ are constants with the same dimension as the viscosity coefficient. For simplicity, let us assume the flow velocity vector,

4 $${\mathbf{v}} = \left( {v_{x} , v_{y} ,v_{z} } \right) = \left( { - {\Omega }y, {\Omega }x,V_{z} } \right),$$

and the director field,

5 $${\mathbf{n}} = \left( {n_{x} , n_{y} ,n_{z} } \right) = \left( {\sin \left( {q_{0} z - \omega t} \right), \cos \left( {q_{0} z - \omega t} \right),0} \right),$$

where the linear flow in the *z*-direction at the velocity of $V_{z}$ is introduced together with the rotational flow at the angular velocity of ${\Omega }$. The positive ${\Omega }$ defines the flow with the left-hand rotation, and the negative, the right-hand rotation. Equation (5) describes the director field of a single-helix structure winding along the *z*-axis with a pitch wavenumber of $q_{0}$, whose value is set to be positive to produce a left-handed helix in accordance with the sample used in the above experiments. The director field rotates at the angular velocity of $\omega$. Moreover, the positive $\omega$ defines the left-hand rotation of the director. Substituting Eqs. (4) and (5) into Eqs. (1)–(3) yields

6 $$W = \frac{1}{2}\gamma_{1} \left( {q_{0} V_{z} - \omega + {\Omega }} \right)^{2} .$$

For simplicity, we assume that the spatial gradients of $V_{z} , \omega$ and ${\Omega }$ are sufficiently small, so the dissipation due to these gradients may be negligible. Owing to this assumption, the terms including $\beta_{1}$, $\beta_{2}$, $\beta_{3}$, and $\gamma_{2}$ vanish, because $e_{ij}$ in Eq. (2) becomes zero tensor. Because $\dot{F}$ is also negligible as mentioned above, the time evolution is determined solely by minimising $W$ [Eq. (6)].

Equation (6) is derived from the forth term of Eq. (1), and hence is proportional to $N_{i}^{2}$. Thus, $N_{i}^{2}$ is what should be minimized for the present purpose. $N_{i}$ is given by Eq. (3) in which the first term represents the director rotation, the second the advection of the director, and the third the rotational flow. In the present situation, since the flow along the z direction $V_{z}$ is passing through the helical director alignment, the second term takes a nonzero value and increases $N_{i}^{2}$. To reduce this, the first and the third terms should take nonzero values, which means the appearance of the director rotation and the rotational flow. In such a way, it is concluded that these rotational motions are driven by the migration of molecules in the direction parallel to the helical axis (see Supplementary Video 3). We should consider that the helix along z axis exist even under the homeotropic anchoring condition, as already reported in literature^31–33^.

Here, one may concern the reliability of our assumption on the flow and the director fields (Eqs. (4) and (5)), because there surely exists the incompatibility between the real and ideal situations. For example, the existence of the radial flow is neglected in Eq. (4), and Eq. (5) is not satisfied under the homeotropic anchoring. This is discussed in Supplementary Note, where the dissipation function is calculated with a slightly-generalised model to describe the experimental situation more exactly. At any rate, as a consequence of the discussion, it was confirmed that the application of Eq. (6) is qualitatively valid even under the radial flow and the homeotropic anchoring.

Equation (6) shows that the minimisation of the dissipation function is performed when $V_{z}$ and $\omega - {\Omega }$ take the finite values with the same sign. Hence, the requisite condition is

7 $$\omega - {\Omega } > 0\;\;{\text{for}}\;\;V_{z} > 0,$$

or

8 $$\omega - {\Omega } < 0\;\;{\text{for}}\;\;V_{z} < 0.$$

Each condition conveys the fact that the director rotation and rotational flow are induced under an existence of the flow along the *z*-axis (// the helical axis). At the same time, the rotational sense alters depending on the directionality of the vertical flow, i.e. upward or downward.

On the basis of the above equations, we further discuss the experimental results. Now, we divide the pillar into three regions, Regions-I, II and III, bounded by the cylinders given by the dashed lines in Fig. 6b, regarding the sign (directionality) of the local vertical flow component $V_{z}$ of each region. Then, the flow conditions in these regions are

9 $$V_{z} > 0\;\;{\text{for Region-II}},$$

10 $$V_{z} < 0\;\;{\text{for}}\;{\text{ Region}}  {\text{-I }}\;{\text{and }}\;  {\text{-III}}.$$

Equations (7)–(10) show that ${\Omega }$ is positive in Region-I and negative in Region-II when $\omega = 0$, which is the fixed director condition. In other words, ${\Omega }$ is positive in the region where the radial coordinate $r$ is small (near the central axis of the pillar) and negative where $r$ is relatively large. This finding looks consistent with the tendency of the flow-field measurement for the fixed director field in the Ch LC pillar upon the homogeneous anchoring, as shown in Fig. 3. By contrast, ${\Omega }$ should become positive in Region-III, which is the edge region of the pillar, but this condition was not clearly shown in Fig. 3. This contradiction would be due to the oversimplification of the present model, for example, neglect of the existence of the velocity gradient, which may cause an additional dissipation. In fact, the large velocity gradient should be inhibited in the minimisation of the dissipation function, which may also prohibit a positive ${\Omega }$ in Region-III. In addition, the assumed director field could have a slight incompatibility with the real structure. Particularly, in the present study, the interface between LC and the solvent has a weak anchoring property^16,18,26,27^, which can deform the helical structure around the edge region of the pillar. For these reasons, the rotational torque and behaviour in Region-III may become different from the theoretical consideration.

The situation is more complicated if the director is not fixed ($\omega \ne 0$) under the existence of a double convective flow. Here, the absolute value of $\omega$ is greatly larger than that of ${\Omega }$ ($\left| \omega \right| \gg \left| {\Omega } \right|$), as observed in Fig. 2. Equations (7)–(10) suggest that $\omega$ also changes with ${\Omega }$, depending on the radial coordinate $r$. $\omega$ is expected to be positive in Region-II and negative in Regions-I and -III. However, such a differential rotation of the director may lead to a large director deformation, which hugely increases the elastic free energy *F*. Because this would increase the Rayleighian ${\Re }$, the appearance of the gradient in $\omega$ is considered to be inhibited. Indeed, static director fields are often realised by the balance of the viscous and elastic forces in the LC pillars under inhomogeneous flows, as reported in refs. 26 and 27. In the present experiment, the observed left-handed director rotation ($\omega > 0$) was in fact accompanied by a negligible or no director deformation (Fig. 1), so $\omega$ is almost invariant over $r$.

In such a situation, the rotational direction would be determined solely by the total torque acted in the pillar. Because $\omega > 0$ is the preferred condition for $V_{z} > 0$ in Region-II, the torque in Region-II should be larger and dominate over all regions, including Regions-I and III, where $\omega < 0$ must be preferred according to the formulation. Therefore, a strong frustration may occur in Region-I: the observed $\omega$ is positive, although $\omega$ is preferred to be negative. To reduce this frustration, ${\Omega }$ would take the positive value in Region-I as deduced from Eqs. (8) and (10). This finding is consistent with our experimental result in which a positive ${\Omega }$ appears and decreases monotonically from $r = 0$ to 100 μm, as shown in Fig. 2. However, because the absolute value of $\omega$ is greatly larger than that of ${\Omega }$, $\omega - {\Omega }$ is still positive everywhere in the pillar, contradicting to Eqs. (8) and (10). In such a way, our oversimplified model does not well explain the real situation, particularly the $r$ dependence of ${\Omega }$. To determine the strict relation between the director and flow fields, we need a model with a more precise and practical distribution for the director and flow fields. Accordingly, more detailed observation and measurements must be performed with a higher space and time resolutions. The exact calculation for the viscous dissipation based on the modelling is also necessary.

At last, it would be worth noting the possible thermal effect. It has been theoretically predicted that the direct coupling between the temperature gradient and the director rotation exists in Ch LC^6,11,34^. Of course, we cannot deny the possibility that this direct thermal coupling effect lies in the present phenomenon. However, even for evaluating and discussing this thermal effect, analysis of the coupling effect between the flow and the director fields is necessary as we have discussed in this paper. In this sense, it should be verified, first and foremost, whether the present phenomenon can be described only by the flow-director coupling. Thus, at the moment, the direct thermal coupling effect is eliminated from the scope of the present study.

In the first part of this paper, we have experimentally analysed the differential rotation induced by the temperature gradient in Ch LC pillars. Under the homeotropic surface anchoring condition, the director rotation was clearly observed together with the rotational flow. The flow field mapping experiment revealed that differential rotation was the essential mode of this rotational flow. The differential rotation of the flow field was also found even in the case of the homogeneous surface anchoring condition, in spite of the fixation of the entire director of the pillar. Further analysis for an N LC pillar confirmed an existence of a double convective flow under the temperature gradient, suggesting that the differential rotation in these samples is driven by an inhomogeneous material flow.

In the second part, the discussion based on Onsager’s variational principle was made upon the relationship between the rotational flow and the director rotation observed in the above experimental results. Here, we assumed a single-helix structure embedded in the Ch LC pillar where the material flow is along the helical axis. Dissipation induced by the velocity gradient was neglected for simplicity. On the basis of these assumptions, we attempted to provide a reasonable explanation for the relation between the director rotation and rotational flow. The experimental results are partially explained by our simplified model but not wholly due to the oversimplification. In the future, a proper assumption for the director and flow fields should be necessarily given, practically with a more precise mapping of the flow field.

## Methods

### Sample preparation

We dispersed an N or Ch LC composed of a host nematic LC (E8, Merck Ltd.) and a chiral dopant (S)-2-octyl 4-[4-(hexyloxy)benzoyloxy] benzoate (S811, Tokyo Chemical Industry Co., Ltd.) (for Ch LC only) into an isotropic fluorinated oligomer (PF656, OMNOVA Solutions Inc.). The Ch LC shows a left-handed helical structure at a bulk state. The weight ratio of LC and PF656 was LC:PF656 = 7:3. For the fluorescence photobleaching method, we added a molecular dye (NBD C6-ceramide, Wako Pure Chemical Industries, Ltd.) with a weight fraction of 0.05 wt%.

We made two types of sandwich cells with homeotropic and planar anchoring conditions. For the homeotropic anchoring, we used a glass substrate coated with a fluorinated resin (CYTOP, AGC, Ltd.). For the planar anchoring, we used a polyimide (Al1254, JSR Co., Ltd) and rubbed the coated surface unidirectionally.

## Temperature control and polarised microscopy

Inserting the samples into the sandwich cell by capillary suction, we applied a temperature gradient to the cell in a homemade sample furnace (for more details about the furnace, see ref.^16^). Then, we made observations using a commercial polarising microscope (BH2, Olympus) equipped with a digital camera (EOS Kiss X50, Canon).

### Fluorescence photobleaching method

For the flow-field analysis with the photobleaching method, we used a commercial microscope (BX61, Olympus) and a charge-coupled device camera (Retiga 4000R Fast 1394, Qimaging). A mercury lamp was used for the light source of the photobleaching and fluorescence microscopy. With the use of photomasks with a lattice or line pattern, the fluorescent dye doped in the sample was patterned with local bleaching by strong light illumination. Then, from the time evolution of the fluorescence images, we analysed the flow field. In the thick cells, the surficial flow fields at higher- and lower-temperature sides can be separately measured by simply flipping the sample furnace on the microscope stage. The detailed description of the method is given in Supplementary Method and ref.^13^.

## Supplementary information

Supplementary Video 1.

Supplementary Video 2.

Supplementary Video 3a.

Supplementary Video 3b.

Supplementary Information.

## Data Availability

All data that support the findings in this study are available in the article and in Supplementary Information. Additional information is available from the corresponding author upon reasonable request.
