# Supplementary material for: Differential rotation in cholesteric pillars under a temperature gradient
Source: Sci Rep. 2020 Oct 14;10:17226. doi: 10.1038/s41598-020-73024-0 (PMC7560747; doi:10.1038/s41598-020-73024-0)
Supplement: Supplementary file 5 — Supplementary Information. [file 41598_2020_73024_MOESM5_ESM.pdf]

Differential rotation in cholesteric pillars under a temperature gradient

Jun Yoshioka and Fumito Araoka

Supplementary information

1. Caption for videos

2. Supplementary Method

3. Supplementary Note

1. Caption for videos

Video 1. Movie of the rotational motion in the Ch LC pillar under a temperature gradient (SI1.mp4). The timescale is 5 times faster than real. The experimental condition is the same with Fig.1.

Video 2. Movie of the Ch LC pillar with planar anchoring under a temperature gradient (SI2.mp4). The timescale is 5 times faster than real. The optical set up is the same with that in Fig.1. The cell thickness was 20μm, and averaged temperature was 35°C. The direction of the temperature gradient ∇T was parallel to the paper, and the applied heat flow was ~14.8mW/mm^2. No rotational motion was observed because of the director field was fixed by the planar anchoring.

Video 3. Schematic representation of director rotation and rotational flow driven by unidirectional flow in Ch LC (SI3(a).mp4 and SI3(b).mp4). In these movies, the situation of Eqs. (4) and (5) is described: a linear flow along the helical axis (z axis) of a left-hand single helix structure is assumed. Owing to the flow, each molecule moves along z axis as time passes. The central movie shows the cross-section parallel to z axis. Both left and right-side movies are the cross-section perpendicular to z axis, while the left one is shown in the coordinate system moving with the same velocity with the flow, and the right is in the spatially fixed coordinate. In SI3(a), the director is not fixed, and each molecule translates with their orientations kept. In this case, the director rotation is induced as shown in the right-side movie. On the other hand, in SI3(b) the director is fixed. In this case, each molecule should move with rotating its orientation as shown in the left-side movie. Owing to this rotational motion, the rotational flow is induced. It should be noted that the directions of the director rotation in (a) and the rotational flow in (b) are opposite from each other.

2. Supplementary Method. Detailed description about fluorescence photo-bleaching method.

For the flow-field analysis with the photo-bleaching method, we used a commercial microscope (BX61, Olympus) and a CCD camera (Retiga 4000R Fast 1394, Qimaging). The schematics of the experimental system is shown in Fig. S1. When the sample was irradiated with a strong blue light from the mercury lamp during 3 seconds, the fluorescent dyes were photo-bleached. Here, using a photomask, we bleached the dyes as shown in Fig. S2(a). After the excitation beam was turned off, the whole sample was weakly illuminated by the blue light from the mercury lamp through ND filters. Here, the sample was irradiated through the objective lens just above the cell, and we set the light to be focused on just below the upper cell substrate. In this experiment, we used the cells with thickness 20 or 50μm, and the bleached dyes diffused to the region with radius ~30μm during the measurements as shown in Fig. S2(d). When the sample with the thickness of 50μm was irradiated, the dyes near the upper cell substrate was more bleached rather than those near the lower substrate. The measurement was made under the temperature gradient. Hence, when the upper substrate was cooled or heated, the flow field in the low or the high-temperature side was measured, respectively.

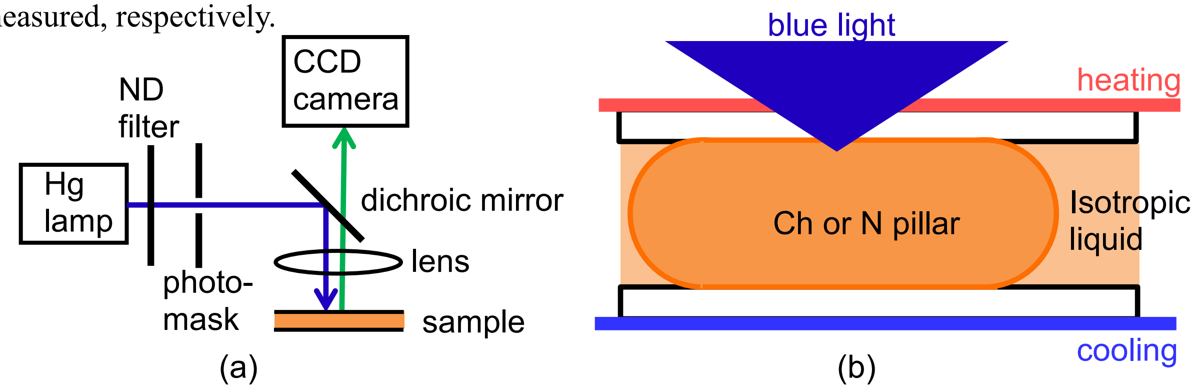

Fig. S1 (a) Schematic image of measurement system. (b) Expanded schematic image near sample cell. In this image, since upper side substrate is heated, the flow-field in the high-temperature side is measured. When the upper side substrate is cooled, the flow-field in low-temperature side is measured.

In this study, we bleached the samples with a lattice or a line pattern. The analysis procedure for the former case is similar to that described in ref. 13; thus, we show the procedure for the latter case. As shown in Figs. 2(a), 3(a) and 4(a) the samples are bleached with a line pattern just after the photo-bleaching. We set the direction perpendicular and parallel to the line pattern x and y axis respectively. In this measurement, we can obtain the flow velocity component along x direction v_x.

In the measurement we obtained the time evolution of the 2-dimensional (2D) fluorescence intensity profiles after the photo-bleaching (I). Normalizing them by the profiles before the photo

bleaching (I_0), we obtained the intensity ratio profiles, I/I_0 . From these 2D profiles, we made 1-dimensional (1D) intensity profiles along the x direction, with averaging along the y direction with a certain length as shown in Fig. S2. Here, the profile should be proportional to the Gaussian function below[13]:

$$\frac{I}{I_0} \propto \frac{1}{\sqrt{1+2Dt/\sigma^2}} \exp\left(-\frac{(x-(x_0+v_x t))^2}{2\sigma^2+4Dt}\right), \quad \text{(S1)}$$

where x_0, D and σ are constants. Eq. (S1) shows that the Gaussian centre is a linear function with respect to the time t, and that its slope corresponds to v_x. The 1D intensity profiles were well fit with Eq. (S1), and we successfully obtained v_x as shown in Figs. S2 (b) and (c). Repeating this procedure with the analysis area changed, we obtained the y dependence of v_x.

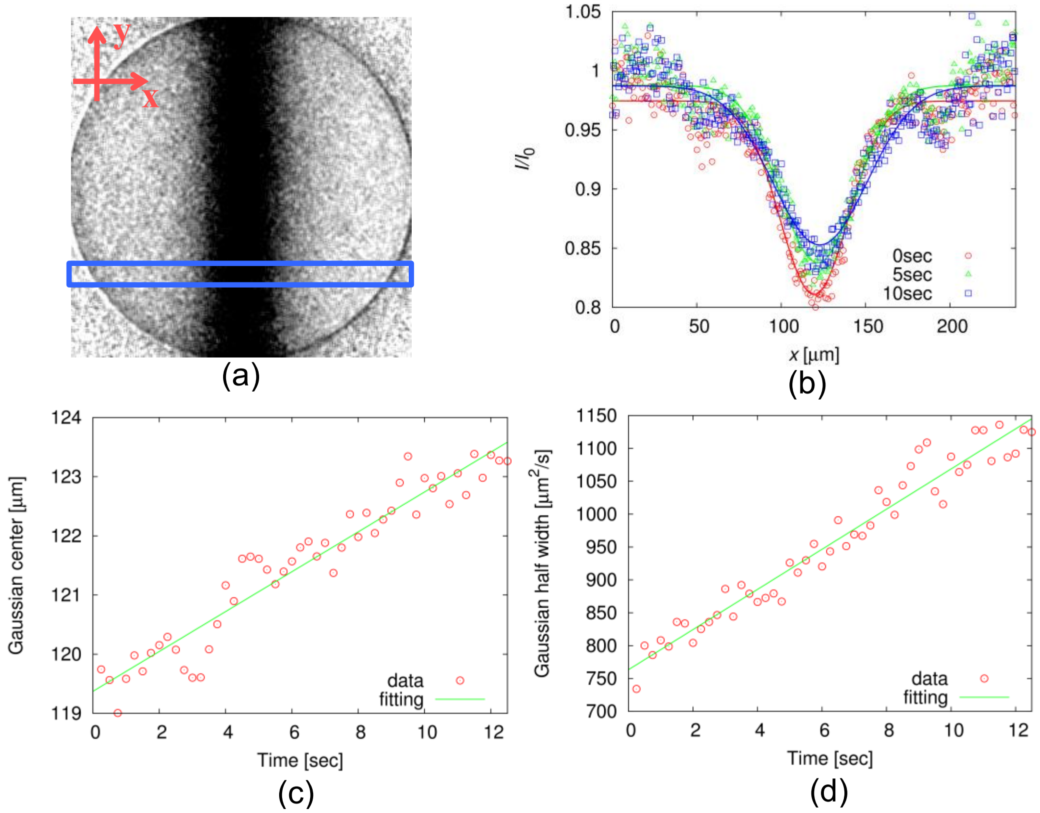

Fig. S2. Procedure for obtaining v_x in fluorescence photo-bleaching method. (a) is the same figure with Fig.2 (a). Using the intensity profiles in the area framed with the blue box, we obtained the 1D intensity profiles along the x direction as shown in (b). The data in (b) are well fitted with the Gaussian of Eq. (S1), and the time evolution of the Gaussian centre is obtained as shown in (c). Here, the slope of the graph in (c) indicates v_x. (d) is a time evolution of the half width of Gaussian, $2\sigma^2+4Dt$ (see, Eq. (S1)). From the fitting with the linear function, the diffusion constant D is obtained as 7.6μm^2/sec. In addition, it can be estimated the dyes diffuses to the region with the radius ~30μm during the measurements.

Setting the coordinate origin to be the pillar centre, we considered that the observed 2D flow would be the linear combination of the radial and rotational flows. Thus, v_x can be described as

$$v_x = v_r \cos\phi - v_\phi \sin\phi \text{, (S2)}$$

where ϕ is azimuthal coordinate, and v_r and v_ϕ are the flow velocity component of the radial and azimuthal direction respectively. When the centre of the bleached line pattern goes through the origin, the radial flow is not observed because $\phi = \pm\pi/2$. In this case, the angular velocity of the rotational flow can be obtained by the relation:

$$\Omega = v_\phi / r = \begin{cases} -v_x/r & \text{for} \quad y > 0 \\ v_x/r & \text{for} \quad y < 0 \end{cases} . \quad \text{(S3)}$$

When the rotational flow can be neglected in Eq. (S2), v_r is obtained by the relation:

$$v_r = \frac{v_x}{\cos\phi} . \quad \text{(S4)}$$

3. Supplementary Note.

Calculation of dissipation function with a slightly generalized model. In the dissipation function W in Eq. (6), the calculation was performed under the assumption of no radial flow ($v_r = 0$). In this note, we calculate W without this assumption. Using a cylindrical coordinate (r, ϕ, z), the flow field is assumed to be

$$\mathbf{v} = (v_r, v_\phi, v_z) = (V_r, \Omega r, V_z) \quad . \quad \text{(S5)}$$

Equation (S5) is consistent with Eq. (4) when $V_r = 0$. In addition, we set the trial function of the director as shown below:

$$\mathbf{n} = (n_r, n_\phi, n_z) = (\sin\phi_n \sin\theta_n(r,z), \cos\phi_n \sin\theta_n(r,z), \cos\theta_n(r,z)) , \quad \text{(S6)}$$

$$\phi_n = q_0 z - \omega t + \phi . \quad \text{(S7)}$$

In Eq. (S6), $\theta_n(r,z)$ represents the polar angle of the director, and it is assumed to be a function of the r and z coordinates. When $\theta_n = 0$, the director aligns along z-axis; thus, if we use the trial function θ_n which is zero at the cell substrate, homeotropic anchoring condition is satisfied. (An example is shown in Fig. S3.) On the other hand, when $\theta_n = \pi/2$ not depending on the coordinates, Eq. (S6) is consistent with Eq. (5).

Substituting Eqs. (S5)–(S7) into Eqs. (1)–(3) yields

$$\begin{aligned} W = {} & \frac{\gamma_1}{2}\left[(q_0 V_z - \omega + \Omega)^2 \sin^2\theta_n + \left(V_r \frac{\partial \theta_n}{\partial r} + V_z \frac{\partial \theta_n}{\partial z} \right)^2 \right] \\ & - \frac{\gamma_2 V_r}{2r}\left[(q_0 V_z - \omega + \Omega)\sin^2\theta_n \sin 2\phi_n - \left(V_r \frac{\partial \theta_n}{\partial r} + V_z \frac{\partial \theta_n}{\partial z} \right) \sin 2\theta_n \cos^2\phi_n \right] , \quad \text{(S8)} \\ & + \frac{V_r^2}{2r^2}\left[\beta_2 + \beta_3 \sin^2\theta_n \cos^2\phi_n + \beta_1 \sin^4\theta_n \cos^4\phi_n \right] \end{aligned}$$

where the terms related to the spatial gradients of V_r, V_z, ω and Ω are neglected as well as in the calculation process of Eq. (6). Here, neglecting the dependence of V_r, V_z, ω and Ω on the azimuthal coordinate ϕ, we integrate Eq. (S8) with ϕ. Consequently, we obtain

$$W_\phi = \int_0^{2\pi} W d\phi = \pi\gamma_1 \left[(q_0 V_z - \omega + \Omega)^2 \sin^2\theta_n + \left(V_r \frac{\partial\theta_n}{\partial r} + V_z \frac{\partial\theta_n}{\partial z} \right)^2 \right]$$
$$+ \frac{\pi\gamma_2 V_r}{2r} \left(V_r \frac{\partial\theta_n}{\partial r} + V_z \frac{\partial\theta_n}{\partial z} \right) \sin 2\theta_n + \frac{\pi V_r^2}{r^2} \left[\beta_2 + \frac{\beta_3}{2}\sin^2\theta_n + \frac{3\beta_1}{8}\sin^4\theta_n \right]. \quad \text{(S9)}$$
$$= \pi\gamma_1 (q_0 V_z - \omega + \Omega)^2 \sin^2\theta_n + W_{\phi 0}(V_r, V_z, \theta_n)$$

The dependence of Eq. (S9) on ω and Ω is consistent with Eq. (6) in the main text. Therefore, the qualitative discussion based on Eq. (6) is possible even when V_r is not zero and θ_n depends on the coordinates r and z.

Concerning on the differences between the experimental and assumed flows and director fields. One may wonder the differences between the experimental situations and the assumptions of the flow and the director fields in the main text. As for the flow field, the existence of the radial flow V_r is neglected in Eq. (4). As for the director field, Eq. (5) is satisfied under the homogeneous anchoring condition, while not under the homeotropic anchoring. We also calculated the dissipation function with using a slightly generalized model, where V_r exists and the director field can satisfy the homeotropic anchoring condition, as described above. Consequently, it was found that the dependence of the dissipation function of ω and Ω is the same with Eq. (6) in the main text when the helix along the z-axis is formed, under the assumptions that V_r, V_z, ω, and Ω are independent of the azimuthal coordinate ϕ and their spatial gradients are sufficiently small. As the formation of the helix along the cell normal (z-direction) under the homeotropic anchoring condition has been observed as reported in refs. 31–33, it is reasonable to consider that the Ch LC pillar in Figs.1 and 2 also has the helix along z-direction. Thus, we consider that qualitative discussion based on Eq. (6) in the main text is applicable even for the case of the homeotropic anchoring under the existence of V_r. On the other hand, we should also note that the assumed director field does not completely describe the POM images obtained in the Ch LC pillar under the homeotropic anchoring condition. In Eq. (5) in the main text and Eq. (S6) in Supplementary Note, the director is assumed to be uniform in the plane parallel to the substrate. Although still we see ununiformity in the POM images of Figs. 1(a)–(c), where a point defect and director distortion were observed, the present model qualitatively explains the phenomenon.

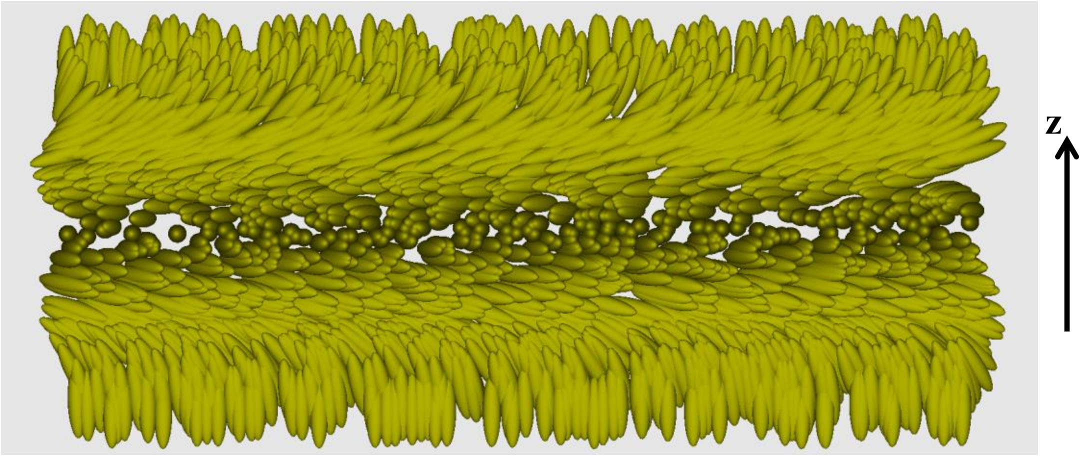

Fig. S3. An example of the director field under homeotropic anchoring. Setting the polar angle as $\theta_n = \pi(1-4z^2/d^2)/2$ in Eq. (S6), we obtain this figure. The cell substrates are in the plane $z = \pm d/2$, where the director aligns along z axis ($\theta_n = 0$). This type of director field has already been reported in refs. 31–33.
